# Supplementary material for: Molecular Characteristics and Polymorphisms of Buffalo (Bubalus bubalis) ABCG2 Gene and Its Role in Milk Fat Synthesis
Source: Animals (Basel). 2023 Oct 9;13(19):3156. doi: 10.3390/ani13193156 (PMC10571847; doi:10.3390/ani13193156)
Supplement: Supplementary file 1 [file animals-13-03156-s001.zip › Supplementary figures.pdf]

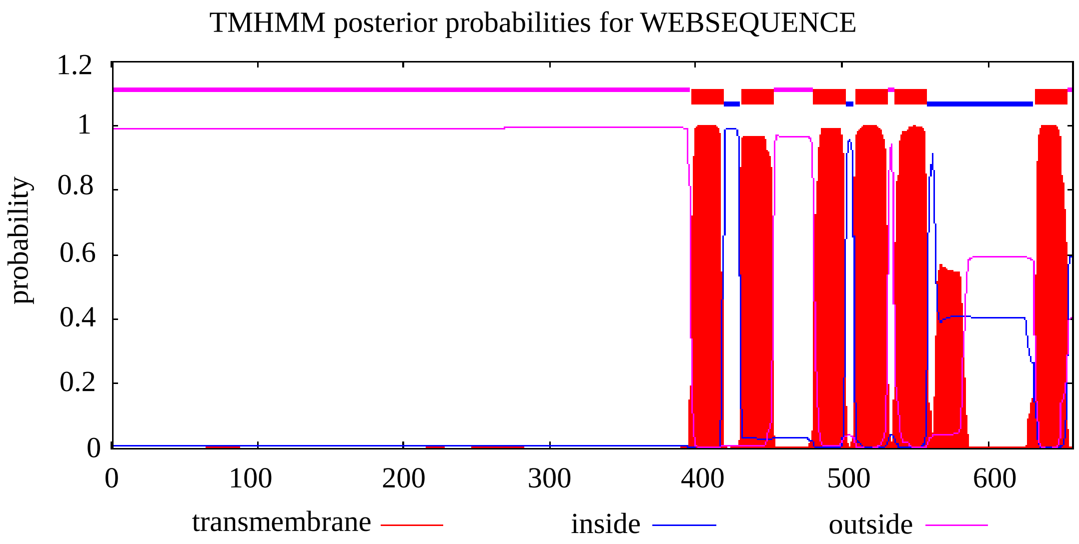


**Figure S1.** Predicted transmembrane helices in buffalo ABCG2

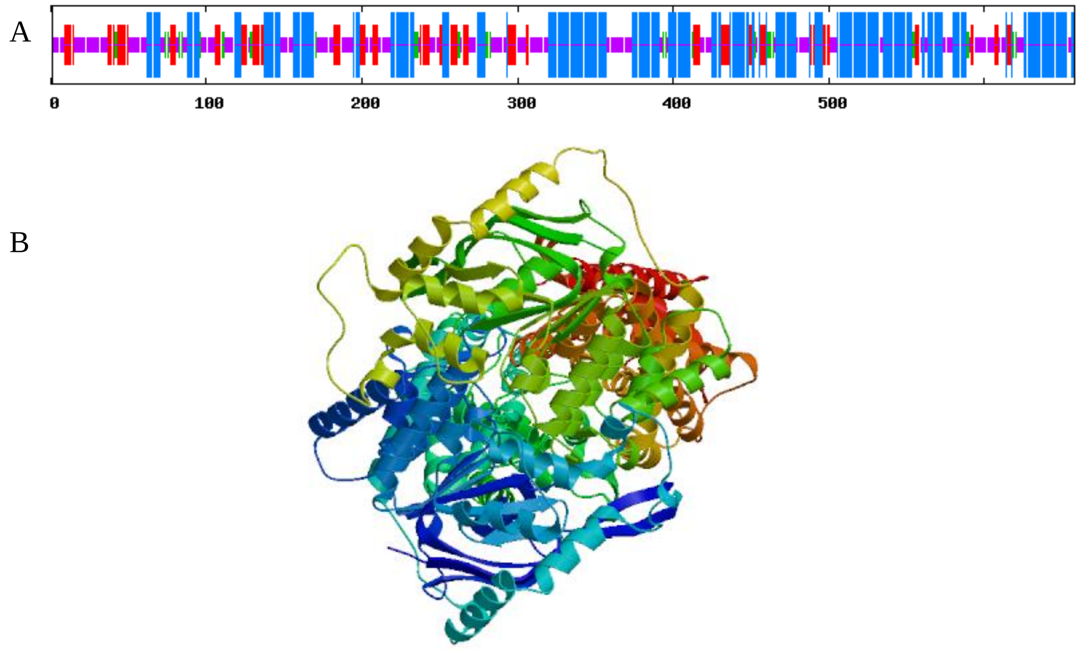


**Figure S2. Structures prediction of buffalo ABCG2.** (A) The secondary structure of ABCG2 protein. (B) The tertiary structure of ABCG2 protein.

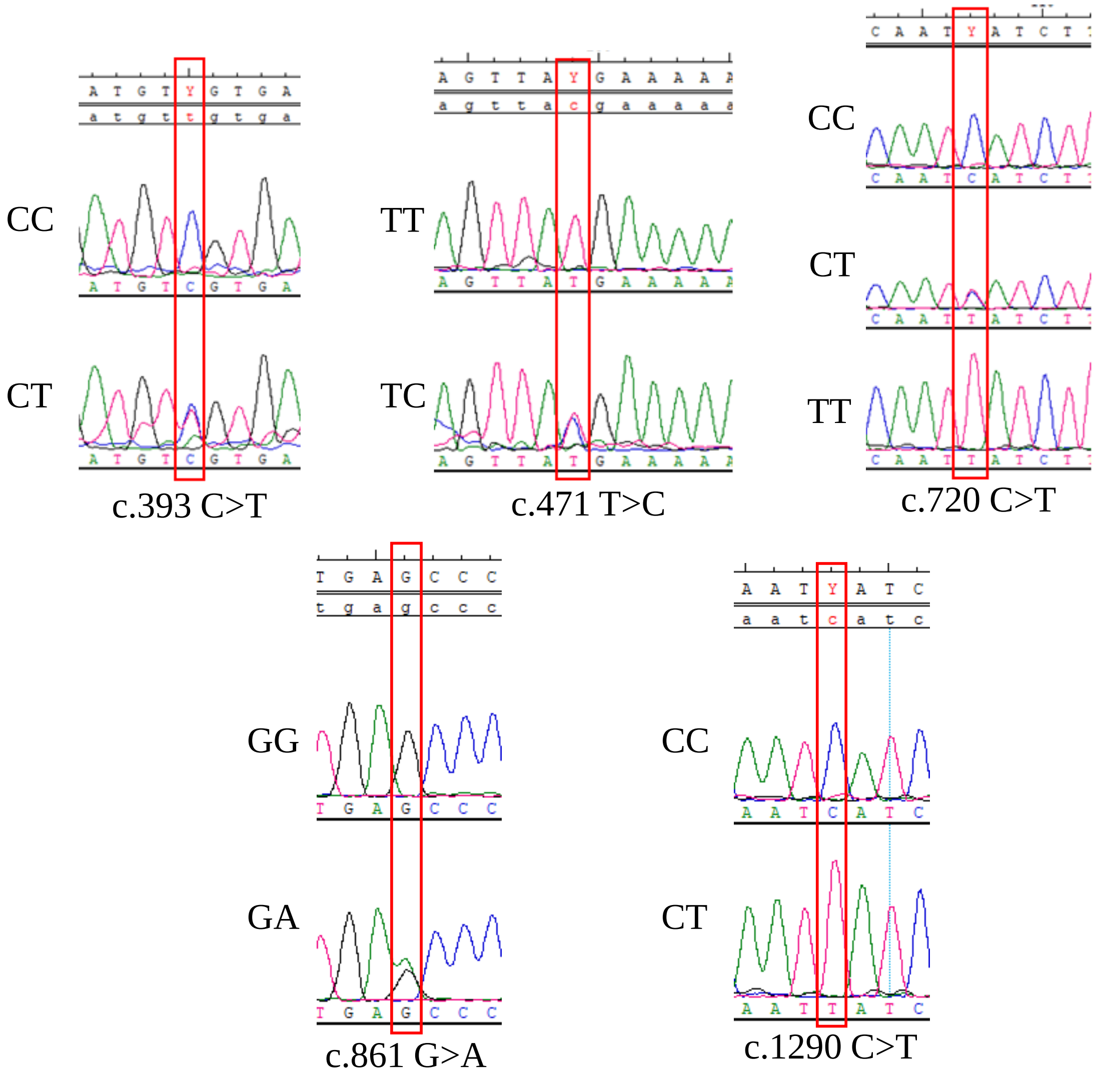


**Figure S3.** Sequencing results indicate polymorphisms in the coding region of buffalo *ABCG2*

```
                                                        111 111 222 333 333 444 444 444 444 444 555 555 555 555 666 666 666 666 666 777 777 777 888 888
                        111 223 333 555 555 889 999 999 222 778 001 777 999 000 333 666 677 888 001 444 666 888 333 455 666 888 889 112 888 999 000 233
                    456 345 890 456 234 567 890 456 789 789 890 890 678 123 012 012 678 901 456 890 789 234 345 789 901 789 567 890 890 789 678 567 901
Buffalo_hap1        CTC TCA GAG TCT AAA ACC CTC ACA TTA AAC ACA AAT GTG GTC GGA TTC AGT TAT CGG TTA CAG GTG AGG GAG GGC GCA CTC CTG ATC TCA CTA CAT GGT
Buffalo_hap2        ... ... ... ... ... ... ... ... ... ... ... ... ... ... ... ... ... ... ... ... ... ... ... ... ... ... ... ... ... ... ... ... ...
Buffalo_hap3        ... ... ... ... ... ... ... ... ... ... ... ... ... ... ... ... ... ... ... ... ... ... ... ... ... ... ... ... ... ... ... ... ...
Buffalo_hap4        ... ... ... ... ... ... ... ... ... ... ... ... ... ... ... ... ... ... ... ... ... ... ... ... ... ... ... ... ..T ... ... ... ...
Buffalo_hap5        ... ... ... ... ... ... ... ... ... ... ... ... ... ... ... ... ... ..C ... ... ... ... ... ... ... ... ... ... ... ... ... ... ...
Buffalo_hap6        ... ... ... ... ... ... ... ... ... ... ... ... ... ... ... ... ... ..C ... ... ... ... ... ... ... ... ... ... ..T ... ... ... ...
Buffalo_hap7        ... ... ... ... ... ... ... ... ... ... ... ... ... ..T ... ... ... ... ... ... ... ... ... ... ... ... ... ... ... ... ... ... ...
Cattle NC_040081    ... ..T ... ... ... CT. ..G ... ... ... ... ... ... ..T ... ... ... ..C ... ... ... ... ... ... ... ... ... ... ... ... ... ..C ...
Cattle XM_027544109 ... ..T ... ... ... CT. ..G ... ... ... ... ... ... ..T ... ... ... ..C ... ... ... ... ... ... ... ... ... ... ... ... ... ..C ...
Cattle BT030709     ... ..T ... ... ... CT. ..G ... ... ... ... ... ... ..T ... ... ... ..C ... ... ... ... ... ... ... ... ... ... ... ..G ..C ..C ..G
Cattle NC_037333    ... ..T ... ... ... CT. ..G ... ... ... ... ... ... ..T ... ... ... ..C ... ... ... ... ... ... ... ... ... ... ... ..G ..C ..C ..G
Cattle XM_024993321 ... ..T ... ... ... CT. ..G ... ... ... ... ... ... ..T ... ... ... ..C ... ... ... ... ... ... ... ... ... ... ... ..G ..C ..C ..G
Cattle NM_001037478 ... ..T ... ... ... CT. ..G ... ... ... ... ... ... ..T ... ... ... ..C ... ... ... ... ... ... ... ... ... ... ... ..G ..C ..C ..G
Cattle XM_027544112 ??? ??? ??? ??? ??? ??? ??? ??? ??? ??? ??? .G. ... ..T ... ... ... ..C ... ... ... ... ... ... ... ... ... ... ... ... ... ..C ...
Zebu NC_032655      ... ..T ... ... ... CT. ..G ... ... ... ... ... ... ..T ... ... ... ..C .A. ... ... ... ... ... ... ... ... ... ... ..G ..C ..C ..G
Zebu XM_019962483   ... ..T ... ... ... CT. ..G ... ... ... ... ... ... ..T ... ... ... ..C .A. ... ... ... ... ... ... ... ... ... ... ..G ..C ..C ..G
Yak NW_005393912    ... ..T ... ... ..G CT. ..G ... ... ... ... ... ... ..T ... ... ... ..C ... ... ... ... ... ... ... ... ... ... ... ... ... ... ...
Yak XM_005897791    ... ..T ... ... ..G CT. ..G ... ... ... ... ... ... ..T ... ... ... ..C ... ... ... ... ... ... ... ... ... ... ... ... ... ... ...
Bison NW_011495025  ... ..T ... ... ... CT. ..G ... ... ... ..T ... ..T ..T ... ..T ... ..C ... ... ... ... ... ..A ... ... ... ... ... ... ... ... ...
Bison XM_010860182  ... ..T ... ... ... CT. ..G ... ... ... ..T ... ..T ..T ... ..T ... ..C ... ... ... ... ... ..A ... ... ... ... ... ... ... ... ...
Sheep XM_042251078  T.. ..T ... ..C ... C.. ..G ..G ..G ... ... ... ... ... ..G ... .A. ..C ... ... ... ..A ..A ... ..T ... ..T T.. ... ... ... ... ...
Sheep NM_001078657  T.. ..T ... ..C ... C.. ..G ..G ..G ... ... ... ... ... ..G ... .A. ..C ... ... ... ... ..A ... ..T ... ..T T.. ... ... ... ... ...
Sheep EE760780      ??? ??? ??? ??? ??? ??? ??? ??? ??? ??? ??? ??? ??? ??? ??? ??? ??? ??? ??? ??? ??? ??? ??? ??? ??? ??? ??? ??? ??? ??? ??? ??? ???
Sheep CU654363      ??? ??? ??? ??? ??? ??? ??? ??? ??? ??? ??? ??? ??? ??? ??? ??? ??? ??? ??? ??? ??? ??? ??? ??? ??? ??? ??? ??? ??? ??? ??? ??? ???
Sheep CU653954      ??? ??? ??? ??? ??? ??? ??? ??? ??? ??? ??? ??? ??? ??? ??? ??? ??? ??? ??? ??? ??? ??? ??? ??? ??? ??? ??? ??? ??? ??? ??? ??? ???
Sheep CU654043      ??? ??? ??? ??? ??? ??? ??? ??? ??? ??? ??? ??? ??? ??? ??? ??? ??? ??? ??? ??? ??? ??? ??? ??? ??? ??? ??? ??? ??? ??? ??? ??? ???
Goat DQ904356       ... ..T ..A .G. ... C.. ..G ..G ..G G.. ... ... ... ... ..G ... .A. ..C ... C.. ..A ... ..A ... ... ..G ..T T.. ... ... ... ... ...
Goat NM_001285707   ... ..T ... ... ... C.. ..G ..G ..G G.. ... ... ... ... ..G ... .A. ..C ... C.. ..A ... ..A ... ... ..G ..T T.. ... ... ... ... ...
Goat NC_030813      ... ..T ... ... ... C.. ..G ..G ..G G.. ... ... ... ... ..G ... .A. ..C ... C.. ..A ... ..A ... ... ..G ..T T.. ... ... ... ... ...

                                                                        111 111 111 111 111 111 111 111 111 111 111 111 111 111 111 111 111 111 111
                    888 888 888 888 888 999 999 999 999 999 999 999 999 999 000 000 000 000 000 000 011 111 111 111 111 111 222 222 222 222 222 222 333
                    334 444 444 555 566 000 444 444 555 666 666 788 889 999 011 111 777 777 888 999 900 000 001 555 777 899 000 122 333 777 777 889 111
                    890 123 456 345 901 789 012 345 567 123 456 901 890 789 901 567 234 567 456 345 901 234 890 345 456 901 456 901 123 012 678 890 567
Buffalo_hap1        GGA GCC ATT CAC GAG GAT ATA GGT AAC ACC GAA AAA CCA GAA GAG TAC GAT CAG AAT TCA TAC AAG GTC AGG CTG GCT TTA TTC GTT GCA ATT GCC AAT
Buffalo_hap2        ... ... ... ... ... ... ... ... ... ... ... ... ... ... ... ... ... ... ... ... ... ... ... ... ... ... ... ... ... ... ... ..T ...
Buffalo_hap3        ... ... ... ... ..A ... ... ... ... ... ... ... ... ... ... ... ... ... ... ... ... ... ... ... ... ... ... ... ... ... ... ... ...
Buffalo_hap4        ... ... ... ... ... ... ... ... ... ... ... ... ... ... ... ... ... ... ... ... ... ... ... ... ... ... ... ... ... ... ... ... ...
Buffalo_hap5        ... ... ... ... ... ... ... ... ... ... ... ... ... ... ... ... ... ... ... ... ... ... ... ... ... ... ... ... ... ... ... ... ...
Buffalo_hap6        ... ... ... ... ... ... ... ... ... ... ... ... ... ... ... ... ... ... ... ... ... ... ... ... ... ... ... ... ... ... ... ... ...
Buffalo_hap7        ... ... ... ... ... ... ... ... ... ... ... ... ... ... ... ... ... ... ... ... ... ... ... ... ... ... ... ... ... ... ... ... ...
Cattle NC_040081    ... ... ... ... ... ... ... ... ... ..T .C. ... ... ... ... ..T ... ... ..G C.. ... ... ... ... ... ... C.. ... ... ... ..C ... ..C
Cattle XM_027544109 ... ... ... ... ... ... ... ... ... ..T .C. ... ... ... ... ..T ... ... ..G C.. ... ... ... ... ... ... C.. ... ... ... ..C ... ..C
Cattle BT030709     ... ... ..A ... ... ... ... ... ... ... ... ... ... ... ... ..T ... ... ..G C.. ... ... ... ... ... ... C.. ... ... ... ..C ... ..C
Cattle NC_037333    ... ... ..A .G. ... ... ... ... ... ... ... ... ... ... ... ..T ... ... ..G C.. ... ... ... ... ... ... C.. ... ... ... ..C ... ..C
Cattle XM_024993321 ... ... ..A .G. ... ... ... ... ... ... ... ... ... ... ... ..T ... ... ..G C.. ... ... ... ... ... ... C.. ... ... ... ..C ... ..C
Cattle NM_001037478 ... ... ..A .G. ... ... ... ... ... ... ... ... ... ... ... ..T ... ... ..G C.. ... ... ... ... ... T.. C.. ... ... ... ..C ... ..C
Cattle XM_027544112 ... ... ... ... ... ... ... ... ... ..T .C. ... ... ... ... ..T ... ... ..G C.. ... ... ... ... ... ... C.. ... ... ... ..C ... ..C
Zebu NC_032655      ... ... ..A ... ... ... ... ... ... ... ... ... ... ... ... ..T ... ... ..G C.. ... ... ... ... ... ... C.. ... ... ... ..C ... ..C
Zebu XM_019962483   ... ... ..A ... ... ... ... ... ... ... ... ... ... ... ... ..T ... ... ..G C.. ... ... ... ... ... ... C.. ... ... ... ..C ... ..C
Yak NW_005393912    ... ... ... ... ... ... ... ... ... ..T ... ... ... ... ... ..T ... ... ..G ... ... ... ... ... ... ... ... ... ... ... ..C ..T ..C
Yak XM_005897791    ... ... ... ... ... ... ... ... ... ..T ... ... ... ... ... ..T ... ... ..G ... ... ... ... ... ... ... ... ... ... ... ..C ..T ..C
Bison NW_011495025  ... ... ... ... ... ... ... ... ... ... ... ... ..G ... ... ..T ... ... ..G ... ... ..A ... ... T.. ... ... ... ... ... ..C ... ..C
Bison XM_010860182  ... ... ... ... ... ... ... ... ... ... ... ... ..G ... ... ..T ... ... ..G ... ... ..A ... ... T.. ... ... ... ... ... ..C ... ..C
Sheep XM_042251078  .A. .A. ... ... ... ..C TC. .A. ..G ... ... ..T T.. ... .G. ..T ..G .G. ..G ... ..T ... A.. .A. ... ... ... ... ... T.. ..C ... ..C
Sheep NM_001078657  .A. .A. ... ... ... ..C TC. .A. ..G ... ... ..C T.. ... .G. ..T ..G .G. ..G ... ..T ... A.. .A. ... ... ... ... ... T.. ..C ... ..C
Sheep EE760780      ??? ??? ??? ??? ??? ??? ??? ??? ??? ??? ??? ??? ??? ??? ??? ??? ??? ??? ??? ??? ??? ??? ??? ??? ??? ... ... .C. ..A T.. ..C ... ..C
Sheep CU654363      ??? ??? ??? ??? ??? ..C TC. .A. ..G ... ... ..T T.. ..T .G. ..T ..G .G. ..G ... ..T ... A.. .A. ... ... ... ... ... T.. ..C ... ..C
Sheep CU653954      ??? ??? ??? ??? ??? ..C TC. .A. ..G ... ... ..T T.. ... .G. ..T ..G .G. ..G ... ..T ... A.. .A. ... ... ... ... ... T.. ..C ... ..C
Sheep CU654043      ??? ??? ??? ??? ??? ..C TC. .A. ..G ... ... ..T T.. ... .G. ..T ..G .G. ..G ... ..T ... A.. .A. ... ... ... ... ... T.. ..C ... ..C
Goat DQ904356       .A. .A. ... ... ... ..C TC. .A. ..G ... ... ..C T.. ... ... ..T ..G ... ..G ... ... ... A.. .A. ... ... ... ... ... T.. ..C ... ..C
Goat NM_001285707   .A. .A. ... ... ... ..C TC. .A. ..G ... ... ..C T.. ... ... ..T ..G ... ..G ... ... ... A.. .A. ... ... ... ... ... T.. ..C ... ..C
Goat NC_030813      .A. .A. ... ... ... ..C TC. .A. ..G ... ... ..C T.. ... ... ..T ..G ... ..G ... ... ... A.. .A. ... ... ... ... ... T.. ..C ... ..C

                    111 111 111 111 111 111 111 111 111 111 111 111 111 111 111 111 111 111 111 111 111 111 111 111 111 111 111 111 111 111 111 111 111
                    333 333 333 333 444 444 555 555 555 555 555 666 666 666 666 666 666 666 666 666 677 777 777 777 777 777 888 888 888 888 888 999 999
                    444 555 666 888 444 888 000 444 555 777 888 011 111 333 555 666 888 999 999 999 900 000 111 222 888 999 000 111 222 333 555 111 666
                    567 123 345 456 123 345 789 012 234 345 567 901 234 345 789 012 789 012 345 678 901 567 456 678 012 567 456 345 567 789 234 567 678
Buffalo_hap1        GAG CTG AAG TAT TTA ATA AAA TTG ATT TCC GCT GTA TCT ACC ATA TTT ACC GTT GTG TCT TGG TCA CAA ATT TTC AAT ACA ACC GCA GGC ACA ATG AAA
Buffalo_hap2        ... ... ... ... ... ... ... ... ... ... ... ... ... ... ... ... ... ... ... ... ... ... ... ... ... ... ... ... ... ... ... ... ...
Buffalo_hap3        ... ... ... ... ... ... ... ... ... ... ... ... ... ... ... ... ... ... ... ... ... ... ... ... ... ... ... ... ... ... ... ... ...
Buffalo_hap4        ... ... ... ... ... ... ... ... ... ... ... ... ... ... ... ... ... ... ... ... ... ... ... ... ... ... ... ... ... ... ... ... ...
Buffalo_hap5        ... ... ... ... ... ... ... ... ... ... ... ... ... ... ... ... ... ... ... ... ... ... ... ... ... ... ... ... ... ... ... ... ...
Buffalo_hap6        ... ... ... ... ... ... ... ... ... ... ... ... ... ... ... ... ... ... ... ... ... ... ... ... ... ... ... ... ... ... ... ... ...
Buffalo_hap7        ... ... ... ... ... ... ... ... ... ... ... ... ... ... ... ... ... ... ... ... ... ... ... ... ... ... ... ... ... ... ... ... ...
Cattle NC_040081    ... ... ... ..C ... ... ..G C.T ..G ... ... ... ... ... ... ... ... ..C ... C.. ... ... ... ... ... ... ... ..G ... ... ..C ... ..G
Cattle XM_027544109 ... ... ... ..C ... ... ..G C.T ..G ... ... ... ... ... ... ... ... ..C ... C.. ... ... ... ... ... ... ... ..G ..C ... ..C ... ..G
Cattle BT030709     ... ... ... ... ... ... ..G C.T ..G ... ... ... ... ... ... ... ... ..C ... C.. ... ... ... ... ... ... ... ..G ..C ... ..C ... ...
Cattle NC_037333    ... ... ... ... ... ... ..G C.T ..G ... ... ... ... ... ... ... ... ..C ... C.. ... ... ... ... ... ... ... ..G ... ... ..C ... ...
Cattle XM_024993321 ... ... ... ... ... ... ..G C.T ..G ... ... ... ... ... ... ... ... ..C ... C.. ... ... ... ... ... ... ... ..G ..C ... ..C ... ...
Cattle NM_001037478 ... ... ... ... ... ... ..G C.T ..G ... ... ... ... ... ... ... ... ..C ... C.. ... ... ... ... ... ... ... ..G ..C ... ..C ... ...
Cattle XM_027544112 ... ... ... ..C ... ... ..G C.T ..G ... ... ... ... ... ... ... ... ..C ... C.. ... ... ... ... ... ... ... ..G ..C ... ..C ... ..G
Zebu NC_032655      ... ... ... ... ... ... ..G C.T ..G ... ... ... ... ... ... ... .S. ..C ... C.. ... ... ... ... ... ... ... ..G ... ... ..C ... ...
Zebu XM_019962483   ... ... ... ... ... ... ..G C.T ..G ... ... ... ... ... ... ... .S. ..C ... C.. ... ... ... ... ... ... ... ..G ..C ... ..C ... ...
Yak NW_005393912    ... ... ..A ... ... ... ..G C.T ..G ... ... ... ... ... ... ... ... ... ... C.. ... ... ... ... ... ... ... ... ... ... ... ... ...
Yak XM_005897791    ... ... ..A ... ... ... ..G C.T ..G ... ... ... ... ... ... ... ... ... ... C.. ... ... ... ... ... ... ... ... ..C ... ... ... ...
Bison NW_011495025  ... ... ... ... ... ... ..G C.T ..G ... ... ... ... ... ... ... ... ... ... C.. ... ... ... ... ... ... ... ... ... ... ... C.. ...
Bison XM_010860182  ... ... ... ... ... ... ..G C.T ..G ... ... ... ... ... ... ... ... ... ... C.. ... ... ... ... ... ... ... ... ..C ... ... C.. ...
Sheep XM_042251078  ... ... ... ... ... ... ... ..T ..G ..G ..C ..G ..C ... ... ... ... A.. .G. G.. ... ..G ..G ..C ... ..C G.. ... ... ..T ... ... ...
Sheep NM_001078657  ... ... ... ... ... ... ... ..T ..G ..G ..C ..G ..C ... ... ... ... A.. .G. G.. ... ..G ..G ..C ... ..C G.. ... ... ..T ... ... ...
Sheep EE760780      ... ... ... ... ... ... ... ..T ..G ..G ..C ..G ..C ... ... ... ... A.. .G. G.. ... ..G ..G ..C ... ..C G.. ... ... ..T ... ... ...
Sheep CU654363      ... T.. ... ... ... ... ... ..T ..G ..G ..C ..G ..C ... ... ... ... A.. .G. G.C ... ..G ..? ??? ??? ??? ??? ??? ??? ??? ??? ??? ???
Sheep CU653954      ... ... ... ... ... ... ... ..T ..G ..G ..C ..G ..C .A. .A. ... ..A T.G .G. G.. G.. ..G .?? ??? ??? ??? ??? ??? ??? ??? ??? ??? ???
Sheep CU654043      ... ... ... ... ... ... ... ..T ..G ..G ..C ..G ..C ... ... ... ... A.. .G. G.. ... ..G ..? ??? ??? ??? ??? ??? ??? ??? ??? ??? ???
Goat DQ904356       ..A ... ... ... ..G ..T ... ..T ..G ..G ..C ... ..C ... ... ..C ... A.. .G. G.. ... ..G ..G ..C ..T ..C G.. ... ... ..T ... ... ...
Goat NM_001285707   ..A ... ... ... ..G ... ... ..T ..G ..G ..C ... ..C ... ... ... ... A.. .G. G.. ... ..G ..G ..C ..T ..C G.. ... ... ..T ... ... ...
Goat NC_030813      ..A ... ... ... ... ... ... ..T ..G ..G ..C ... ..C ... ... ... ... A.. .G. G.. ... ..G ..G ..C ..T ..C G.. ... ... ..T ... ... ...
```

**Figure S4.** Nucleotide differences of the *ABCG2* haplotype sequences among some species of Bovidae. Number represents the position of coding region. Dots (.) denote identity with the Buffalo_hap1. Nucleotide substitutions are denoted by different letters. Missing information is denoted by a question mark (?).
