# Supplementary material for: Molecular Characteristics and Polymorphisms of Buffalo (Bubalus bubalis) ABCG2 Gene and Its Role in Milk Fat Synthesis
Source: Animals (Basel). 2023 Oct 9;13(19):3156. doi: 10.3390/ani13193156 (PMC10571847; doi:10.3390/ani13193156)
Supplement: Supplementary file 1 [file animals-13-03156-s001.zip › Supplementary tables.pdf]

Table S1. Primers information used for qPCR investigated in this study

Gene	Accession number	Primer sequences (5′-3′)	Amplification efficiency
GAPDH	XM_006065800	F: ATGGAGAAGGCTGGGGCTCA<br>R: GCAGGAGGCATTGCTGACAA	2.058
RPS23	XM_006059350	F: ACCGACGAGACCAGAAGT<br>R: CTCCAGGAATGTCACCAA	2.012
ACTB	NM_001290932	F: TGGGCATGGAATCCTG<br>R: GGCGCGATGATCTTGAT	2.032
AGPAT6	NM_001290846	F: CTTTGCGTGGGCTACCTTG<br>R: TCTTGGTCACCTCGTCGTC	2.032
PPARG	XM_006077448	F: GCTCCAAGAGTACCAAAGTG<br>R: GTCCTCCTGAAGAAACCCTT	2.096
SREBF1	KU517672	F: GCACCGAGGCCAAGTTGAATAA<br>R: CAGGTCCTTCAGCGATTTGCTT	2.041
SREBF2	XM_006068914	F: GCCAAGATGCACAAGTCTGGTGTT<br>R: TGCCCTTCAGGAGCTTGCTCT	2.099
INSIG1	JX853922	F: ACGTTCAGCTCTCCTTGACATT<br>R: CTGTCGTCCTATGTTTCCCAC	2.091
INSIG2	NM_001290944	F: AGCCAGGGAGGGAAATGACCAT<br>R: GCCTCTGCCTGTGGGTTACTCA	2.067
PPARGC1A	NM_177945	F: CCACCGAGAATGAGGCTAGTCCTT<br>R: TTGACAAATGCTCTTCGCTTTATTGCTCCA	2.089
FASN	XM_006061793	F: GGGCTCCACCACCGTGTTCCA<br>R: GCTCTGCTGGGCCTGCAGCTG	2.053
ACC	XM_025281121	F: CCTCTTCAGACAGGTTCAAGC<br>R: TTCACCGCACACTGTTCC A	1.968
SCAP	XM_025272558	F: CCATGTGCACTTCAAGGAGGA<br>R: TGTCGATCTTGCGTGTGGAG	1.972
ABCG2	XM_006042277	F: AGCAAATATCAATGGAGTCA<br>R: CGACATCATCTTGTACCACATA	2.062

Table S2. Primer information for polymorphism identification

	Primer sequence (5'-3')	Product length (bp)	Annealing temperature (°C)
Exon 2	F: TTTACCTGGACTATCAAC<br>R: ATGTCAATGAACCTACTTA	512	50.0
Exon 3	F: TTGTGCTTTTTTTTCTATC<br>R: CCAAATGTCAAATCTCTAT	304	45.0
Exon 4	F: GCTAAGAGGTAAAGCCTG<br>R: GCGGGTATAAAGAAAAGTTG	307	48.5
Exon 5	F: AGATATACTACAGAGTGATG<br>R: CTTGACCATACATATTATAG	325	47.0
Exon 6	F: GCTGGAACATTTTAGGAG<br>R: CTTACTGATGAATCCCTTGC	330	51.0
Exon 7	F: CAAGTTGAATGAGGAATAA<br>R: AGTGCTCCAAGTGACTC	344	49.0
Exon 8	F: CACCTCCCTCACTCTCTAA<br>R: CTAGTACAACCCATATCAGA	315	49.0
Exon 9	F: AATGAAGGTGCTAGAGTA<br>R: GGAATGGGGATAAATAAA	455	48.9
Exon 10	F: TAATAATAAAGGGTGTG<br>R: TAAGAATATGAAAACTCTC	304	44.0
Exon 11	F: GGAAGAAAAATGACCTGTAAATGT<br>R: TCTCAGTCAACACTGGTAAC	264	52.0
Exon 12	F: CTGTATAGCAGGGAGTACATCTG<br>R: ACTTGATTTATAGTTTTGAGAA	310	47.0
Exon 13	F: CCCTGATAGACAGTGTTGGATTA<br>R: CCTTAAAGCAGAGTCTGATGTA	315	49.5
Exon 14	F: GTCCTTAGCAGTATTCACGAGAC<br>R: ACTCTCCTTCATTGTCCTAAA	301	49.5
Exon 15	F: AATTCTTTGGAAACTTTTGTCA<br>R: CCTCAGCCCAGTAGTATT	221	51.0
Exon 16	F: TCTTTAAGGAACAGTGAAT<br>R: CAAGTGAAAAGAAGACAACC	381	47.8

Table S3. Information of shRNA used for knockdown of *ABCG2*

Name of shRNA	Sequences (5'-3')
ABCG2_sh1_F	CCGGGTATTGCAATGGAGCTTATTACTCGAGTAATAAGCTCCATTGCAATACTTTTTG
ABCG2_sh1_R	AATTCAAAAAGTATTGCAATGGAGCTTATTACTCGAGTAATAAGCTCCATTGCAATAC
ABCG2_sh2_F	CCGGGAGGATGTTACCAAGTATTATCTCGAGATAATACTTGGTAACATCCTCTTTTTG
ABCG2_sh2_R	AATTCAAAAAGAGGATGTTACCAAGTATTATCTCGAGATAATACTTGGTAACATCCTC
ABCG2_sh3_F	CCGGGGAAGAATCACGTAGCCTTGGCTCGAGCCAAGGCTACGTGATTCTTCCTTTTTG
ABCG2_sh3_R	AATTCAAAAAGGAAGAATCACGTAGCCTTGGCTCGAGCCAAGGCTACGTGATTCTTCC

Table S4. Haplotype information of buffalo *ABCG2* gene

Haplotype	Accession number	Alleles	Actual frequency	Expected frequency
Buffalo_hap1	ON515705	CTCGC	0.892	0.892
Buffalo_hap2	ON515706	CTCGT	0.010	0.010
Buffalo_hap3	ON515707	CTCAC	0.024	0.024
Buffalo_hap4	ON515708	CTTGC	0.049	0.049
Buffalo_hap5	ON515709	CCCGC	0.010	0.010
Buffalo_hap6	ON515710	CCTGC	0.005	0.005
Buffalo_hap7	ON515711	TTCGC	0.010	0.010
